# Supplementary material for: PV1 Protein from Plasmodium falciparum Exhibits Chaperone-Like Functions and Cooperates with Hsp100s
Source: Int J Mol Sci. 2020 Nov 16;21(22):8616. doi: 10.3390/ijms21228616 (PMC7697860; doi:10.3390/ijms21228616)
Supplement: Supplementary file 1 [file ijms-21-08616-s001.pdf]

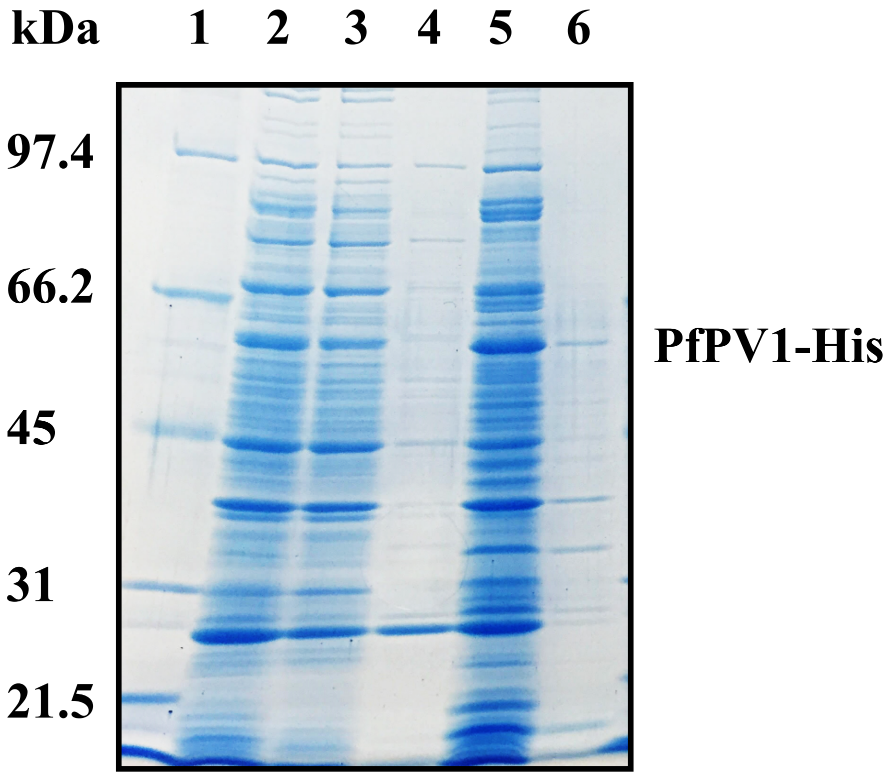

Supplementary Fig. S1 Expression and purification of PfPV1-His

1, Molecular marker; 2, Crude extract; 3, Flow-through;
4, Wash; 5, Elution 1; 6, Elution 2.

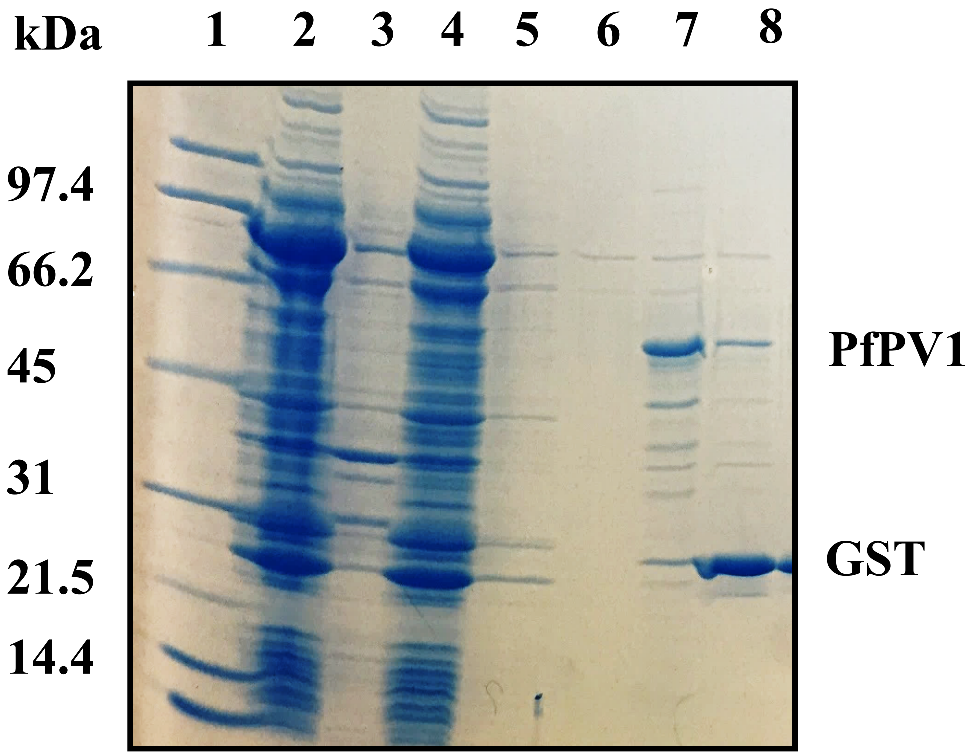

Supplementary Fig. S2 Expression and purification of GST-PfPV1

1, Molecular marker; 2, Crude extract; 3, Precipitant;
4, Flow-through of affinity column; 5, Wash 1 ; 6, Wash 2;
6, Elution by HRV3C protease digestion; 7, Elution by glutathione.

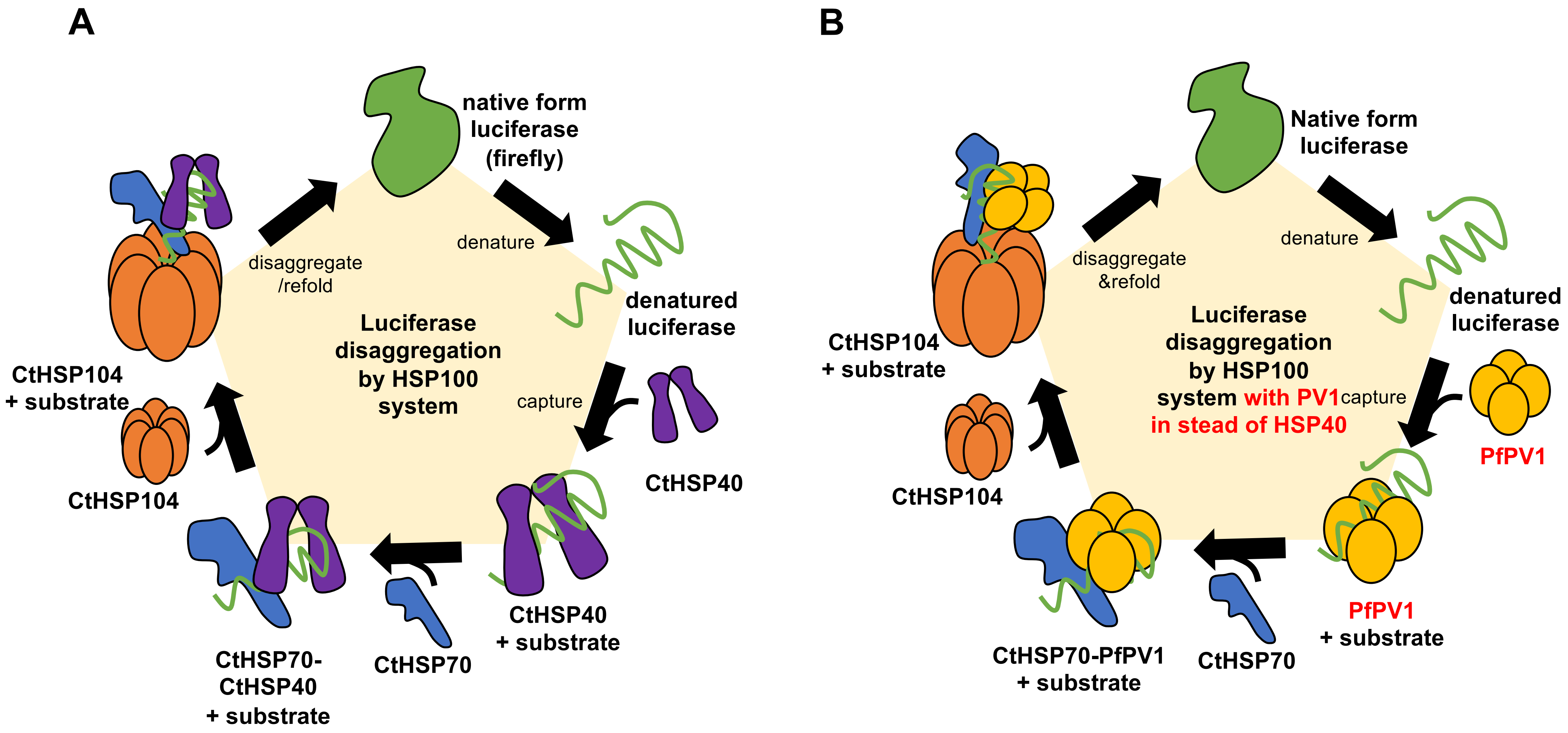

Supplementary Fig. S3
Schematic models for protein disaggregation by CtHsp104 system (A)
and that with the replacement of CtHsp40 by PfPV1 (B)

Supplementary Table S1. Primer sequences

Product	AA range	Fw/Rv	Sequence
PV1_HIS	V23-S452	Fw	CATGccatgGTGGTGGCCCCTAAGAGTG
		Rv	CCGctcgagGCTCGATATTGGTGTGTTTTGATCATTTC
GST_PV1	V23-S452	Fw	CGggatccGTGGTGGCCCCTAAGAGTG
		Rv	CCGctcgagCTAGCTCGATATTGGTGTG
GST_PV1_Strep	V23-S452	Fw	CGggatccGTGGTGGCCCCTAAGAGTG
		Rv	CCGCTCGAGCTATTTTTCAAACTGCGGATGGGACCAGCTCGATATTGGTGTGTTTTG
